# Methodological challenges of measuring brain volumes and cortical thickness in idiopathic normal pressure hydrocephalus with a surface-based approach

**DOI:** 10.3389/fnins.2024.1366029

**Published:** 2024-07-19

**Authors:** Martina Del Giovane, Michael C. B. David, Magdalena A. Kolanko, Anastasia Gontsarova, Thomas Parker, Adam Hampshire, David J. Sharp, Paresh A. Malhotra, Christopher Carswell

**Affiliations:** ^1^UK Dementia Research Institute, Care Research & Technology Centre, Imperial College and the University of Surrey, London, United Kingdom; ^2^Department of Brain Sciences, Imperial College London, London, United Kingdom; ^3^Centre for Neuroimaging Sciences, Institute of Psychiatry, Psychology and Neuroscience, King’s College London, London, United Kingdom; ^4^Department of Neurology, Imperial College Healthcare NHS Trust, London, United Kingdom

**Keywords:** normal pressure hydrocephalus, enlarged ventricles, (NPH), FreeSurfer, brain segmentation, dementia, Alzheimer’s disease (AD)

## Abstract

Identifying disease-specific imaging features of idiopathic Normal Pressure Hydrocephalus (iNPH) is crucial to develop accurate diagnoses, although the abnormal brain anatomy of patients with iNPH creates challenges in neuroimaging analysis. We quantified cortical thickness and volume using FreeSurfer 7.3.2 in 19 patients with iNPH, 28 patients with Alzheimer's disease (AD), and 30 healthy controls (HC). We noted the frequent need for manual correction of the automated segmentation in iNPH and examined the effect of correction on the results. We identified statistically significant higher proportion of volume changes associated with manual edits in individuals with iNPH compared to both HC and patients with AD. Changes in cortical thickness and volume related to manual correction were also partly correlated with the severity of radiological features of iNPH. We highlight the challenges posed by the abnormal anatomy in iNPH when conducting neuroimaging analysis and emphasise the importance of quality checking and correction in this clinical population.

## Introduction

Idiopathic Normal Pressure Hydrocephalus (iNPH) is a neurological condition that affects approximately 0.3–3%, of individuals aged 60 and above (Jaraj et al., 2014). It is characterized by alterations in cerebrospinal fluid dynamics, leading to the enlargement of the ventricles to maintain a stable intracranial pressure (Carswell, 2022). A triad of symptoms; gait apraxia, urinary incontinence, and cognitive deficits, result from this compensatory ventricular expansion, which stretches and distorts the surrounding parenchyma (Carswell, 2022). Therapeutic redirection of cerebrospinal fluid to an area of lower pressure (i.e., shunting) can dramatically improve symptoms (Carswell, 2022).

iNPH occurs in the elderly population in which traditional neurodegenerative diseases are common (Jaraj et al., 2014), and identifying iNPH-specific clinical and imaging features is paramount to being able to distinguish these disorders. The anatomical features of iNPH introduce methodological challenges in neuroimaging analysis. Reduced callosal angle, ventriculomegaly, and disproportionately enlarged subarachnoid space hydrocephalus (DESH) are some of such distinctive features of iNPH seen on brain imaging (Hashimoto et al., 2010). Here, we would like to address potential limitations associated with the use of FreeSurfer,^1^ a software used for the analysis and visualization of brain imaging data, in this specific patient group.

One notable advantage of FreeSurfer is its ability to employ a fully automated pipeline, enabling the segmentation of the brain into regions of interest. It is freely available, widely used and there is extensive experience within the field in implementing it within analysis pipelines aiding reproducibility. FreeSurfer registers the volume with the MNI305 atlas. It performs a surface-based reconstruction of the cortex, which classifies voxels as either white or non-white matter based on voxel intensity and neighbour constraints, and a volume-based stream for volume labelling of each point (voxel) of the brain mask (Dale et al., 1999; Fischl et al., 2002). It derives the white matter surface as the interface between the white and gray matter, and the pial surface as the boundary between the pial and cerebrospinal fluid (CSF). Cortical thickness and volumes can then be quantified in 34 different regions derived from the Desikan-Killiany atlas. This automated process is considerably less laborious and less prone to bias than manual regions of interest segmentation.

Quality control and manual editing can be performed to rectify errors related to skull stripping, grey-white matter segmentation, and intensity normalization.^2^ Several studies have compared the outputs of the FreeSurfer’s pipeline with and without manual edits in groups of healthy adults, individuals with genetic disorders, and severe head injuries and found mixed results (McCarthy et al., 2015; Guenette et al., 2018; Waters et al., 2019). There is also limited research investigating the significance of the manual editing step in clinical populations with extremely abnormal brain morphology, which can impact the registration and segmentation analysis stages.

## Methods

**Figure 1:**
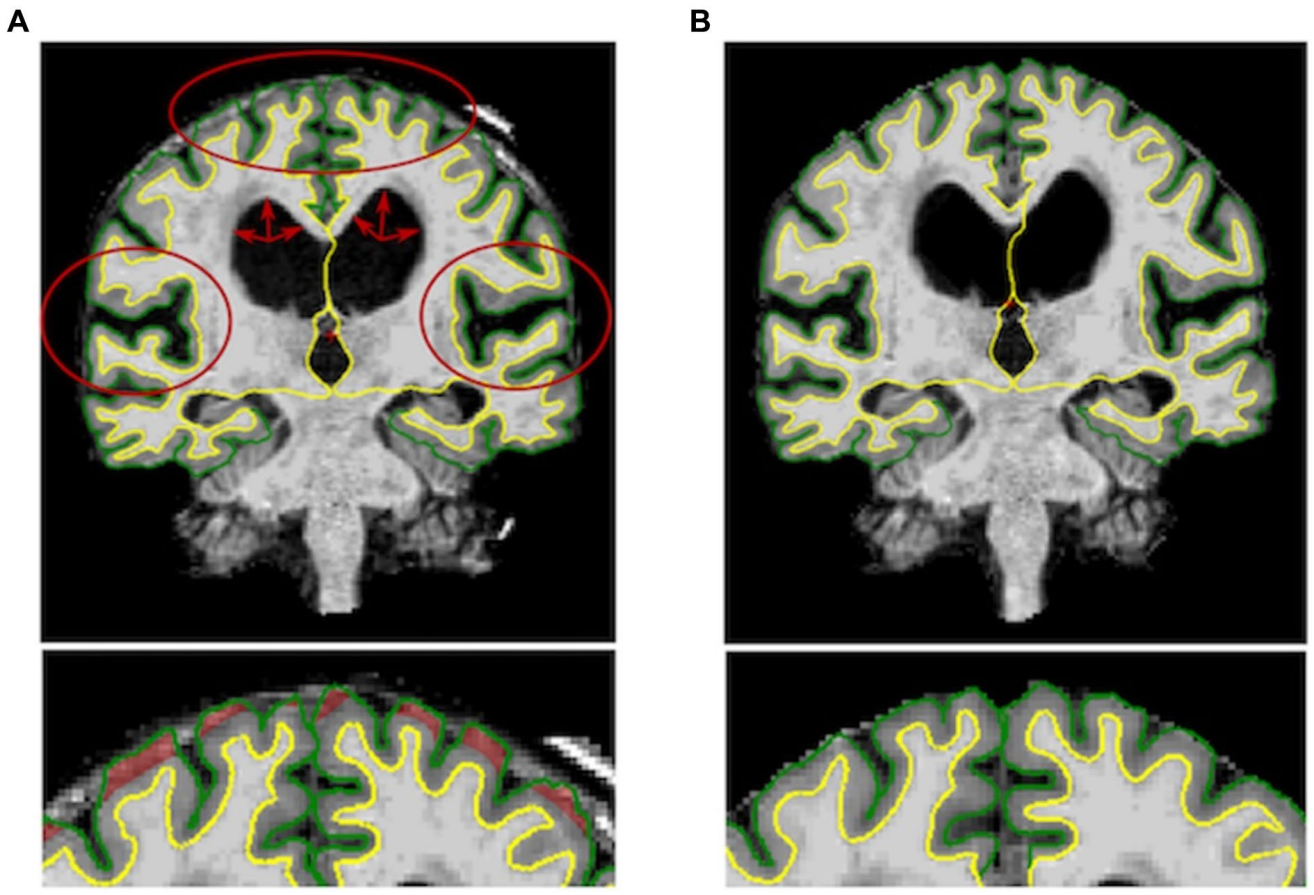
**Left**: Output of the Freesurfer’s recon-all command before the manual editing step for one subject. DESH features (i.e., enlarged ventricles, widened sylvian fissure and tight high convexity) are marked in red. **Right**: Output of the manual editing step for the same subject showing reduced cortical thickness..

We evaluated the importance of manually correcting the segmentation output produced by FreeSurfer 7.3.2^3^ on the MRI scans of 19 patients with iNPH, 28 patients with Alzheimer’s disease, and 30 healthy controls (HC). To improve the readability of the results and reduce multiple comparisons, the 34 regions segmented by FreeSurfer where clustered to derive cortical thickness and volumes for the frontal, temporal, parietal, occipital and cingulate lobes (see Footnote 1). Between-group differences in age and gender were analysed using Kruskal-Wallis test and Chi-Square test, respectively. All scans were visually checked to ensure their quality met appropriate research standards. We then ran the FreeSurfer recon-all command using the -bigventricles flag. Of the 19 iNPH patients, 12 were classified as probable, 4 as possible and 3 as asymptomatic iNPH, as defined by international criteria (Relkin et al., 2005). Among the 16 symptomatic iNPH patients, 15 received a lumbar puncture and had their CSF samples analysed to determine the presence of comorbid AD pathology. Amyloid deposition was detected in two patients. Radiological features of iNPH were assessed and calculated by a neuroradiologist. Participants were scanned on a 3 T Siemens scanner as part of a wider ongoing study run by the UK Dementia Research Institute, Care Research & Technology Centre focused on using sensor technology to monitor behaviours of people living with dementia. We visually inspected each output and performed manual editing when necessary (Figure 1). Wilcoxon signed-ranked tests were used to compare the FreeSurfer’s measurements (volumes and cortical thickness) before and after manual edits while accounting for non-normally distributed data. Between-group differences in these changes were assessed via repeated measures ANOVA, followed by two-tailed t-tests with FDR correction for post-hoc comparisons. Finally, exploratory Spearman correlations were conducted between changes in cortical thickness/volumes pre and post manual correction and radiological features of iNPH (i.e., Radscale score, Evan’s index, callosal angle and DESH score). To assess the potential for rectifying FreeSurfer’s inaccuracies through alternative pre-processing software, we conducted two additional evaluations. First, we integrated the HD-BET tool for skull stripping before executing the FreeSurfer recon-all command. Notably, HD-BET has exhibited superior performance compared to various widely used brain extraction algorithms, even in the presence of brain pathology (Isensee et al., 2019). Additionally, we experimented with running the FreeSurfer recon-all command using a combination of T1 and FLAIR scans.^4^

This study was approved by the Health Research Authority’s London-Surrey Borders Research Ethics Committee (19/LO/0102) and the Health Research Authority’s London-Central Research Ethics Committee (18/LO/0249). All participants gave written and/or electronic consent.

## Results

**Figure 2:**
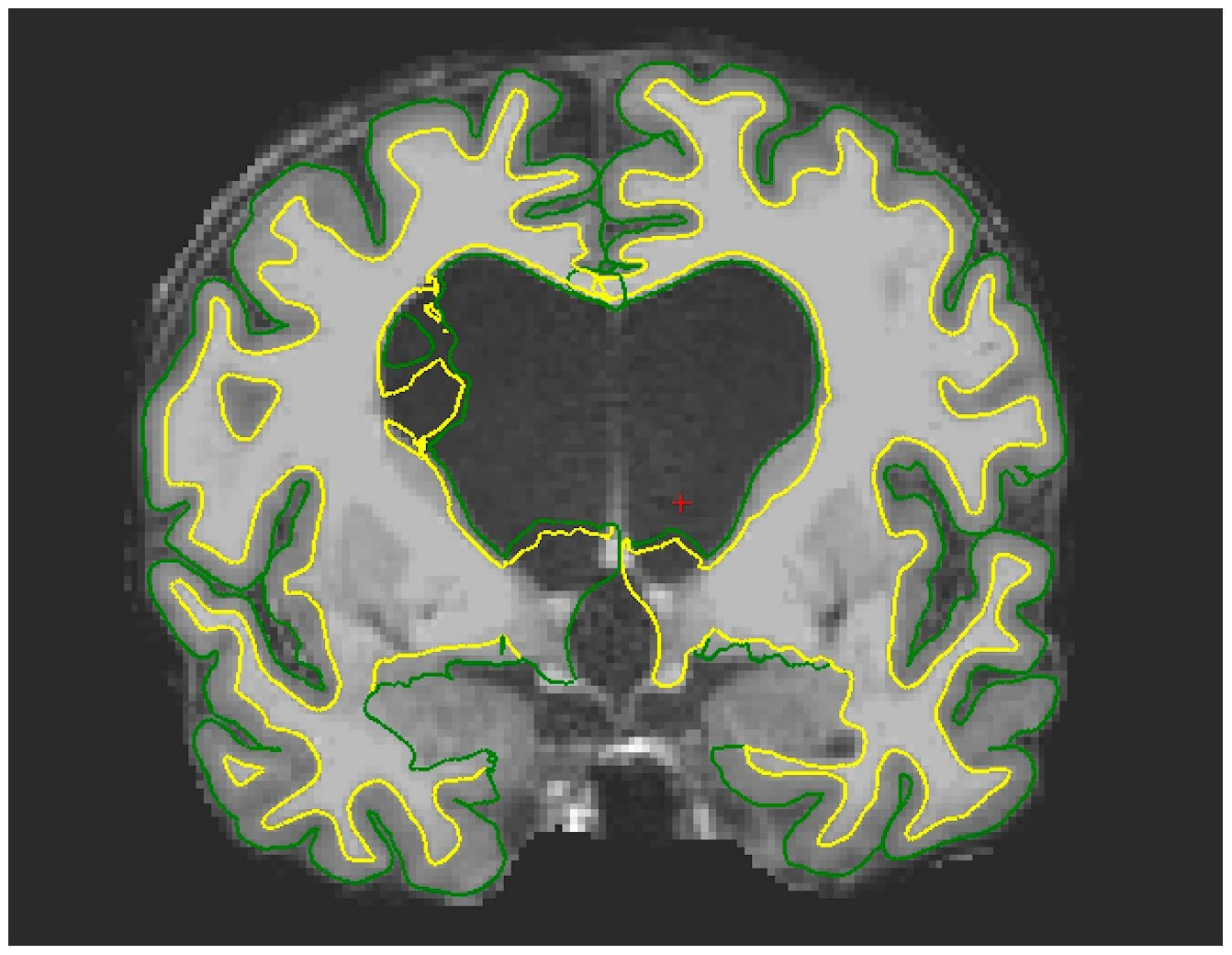
Example of failed segmentation for one iNPH patient. Due to the presence of oedema, the pial and white matter surface are wrongly estimated around the ventricles and extend into the CSF space.

HC (14 females, mean age = 75.58 years, SD = 6.07), AD patients (12 females, mean age 75.25 years, SD =7.64 years) and NPH patients (7 females, mean age = 71.58 years, SD = 5.92 years) did not differ significantly in terms of gender. No significant age difference was found between HC and AD patients. Conversely, iNPH participants were significantly younger than AD and HC (*p* = 0.01). The iNPH patients had a mean Evan’s Index of 0.38 (SD = 0.04), mean callosal angle of 75.7 (SD = 15.83), mean Radscale score of 9.3 (SD = 1.51) and mean DESH score of 7.06 (SD = 1.77). Out of the 19 scans of patients with iNPH, 3 failed the segmentation step (Figure 2) and 15 required extensive manual corrections (Figure 1). Of the 3 patients whose Freesurfer segmentation failed, 2 were asymptomatic. Of the 28 patients with Alzheimer’s disease, one failed the segmentation and 4 required manual corrections. In the HC group, only 2 participants needed manual editing of the segmentation output. No corrections of the white matter surface were required in any study group. In the iNPH group, manual edits aimed to improve the removal of skull and rectify inaccuracies in defining the pial surface, which had extended into the dura and skull. Following manual correction, the parietal, frontal and temporal regions exhibited the most substantial differences; with volume and cortical thickness measures decreasing bilaterally across the group (Table 1). Wilcoxon signed-ranked tests comparing these measurements before and after manual edits did not reach significance, although we may have been underpowered by small participants’ number. Repeated measures ANOVA indicated an effect of group on the delta values of the volumes (*F*_(19,630)_ = 2.84, *p* < 0.001), but not cortical thickness (*p* > 0.05), which suggest potential higher reliability of this measure relative to volumes. Between group differences were observed for the delta values of the frontal, parietal, temporal and cingulate volumes (Table 1). In Supplementary Table S1, we also report the differences in cortical thickness and volumes before and after manual correction for all the 34 individual regions segmented by FreeSurfer and the between-group comparisons of the delta values.

**Table 1:** Values of cortical thickness and volumes before and after manual correction for the 5 main lobes (left and right), and between-group comparisons of the delta values (FDR corrected).

Region	Group	Volume (mm^3^)							Cortical Thickness (mm)						
		Pre correction		Post correction		Delta			Pre correction		Post correction		Delta		
		Mean	std	Mean	std	Mean	std	Group differences of delta	Mean	std	Mean	std	Mean	std	Group differences of delta
Frontal Right	iNPH	27982.59	32251.94	27484.46	31659.98	498.13	889.84	iNPH>AD** iNPH>HC ***	26.52	0.96	26.40	0.89	0.13	0.17	all *p* > 0.05
	AD	72282.74	9094.58	72247.37	9055.27	35.37	202.31		26.01	1.15	26.00	1.16	0.00	0.04	
	HC	77633.17	8287.50	77620.77	8295.22	12.40	47.48		26.32	1.13	26.42	1.18	−0.10	0.72	
Parietal Right	iNPH	23787.64	20635.75	23445.83	20257.78	341.81	706.61	iNPH>AD** iNPH>HC **	11.63	0.57	11.55	0.58	0.08	0.16	all *p* > 0.05
	AD	48385.96	6646.78	48353.67	6666.74	32.30	194.25		10.66	0.56	10.65	0.57	0.01	0.03	
	HC	52999.70	6776.29	52969.13	6758.55	30.57	138.82		11.13	0.53	11.09	0.60	0.04	0.24	
Temporal Right	iNPH	18742.05	20548.49	18548.28	20318.95	193.76	461.20	iNPH>HC*	24.29	1.29	24.15	1.30	0.13	0.35	all *p* > 0.05
	AD	44192.44	6443.66	44122.67	6435.27	69.78	211.15		22.82	1.50	22.83	1.47	0.00	0.06	
	HC	50096.23	5268.73	50099.43	5271.93	−3.20	35.51		24.75	1.01	24.39	1.74	0.36	1.39	
Occipital Right	iNPH	7150.50	4793.46	7164.83	4872.66	−14.33	191.05	all *p* > 0.05	7.86	0.46	7.80	0.49	0.06	0.18	all *p* > 0.05
	AD	24471.63	4030.69	24437.26	4048.02	34.37	165.71		7.69	0.33	7.68	0.34	0.00	0.05	
	HC	25645.37	3492.30	25643.50	3491.76	1.87	18.49		7.78	0.29	7.76	0.31	0.02	0.08	
Cingulate Right	iNPH	3379.86	2485.74	3295.53	2465.91	84.33	234.82	iNPH>AD* iNPH>HC *	8.98	0.45	9.01	0.45	−0.02	0.25	all *p* > 0.05
	AD	8382.74	1091.51	8379.07	1074.23	3.67	46.02		9.24	0.62	9.21	0.62	0.03	0.11	
	HC	8789.83	1348.99	8788.03	1351.10	1.80	12.32		9.35	0.57	9.43	0.54	−0.08	0.30	
Frontal Left	iNPH	28596.39	33052.43	28126.09	32465.30	470.30	989.51	iNPH>HC*	26.63	0.74	26.52	0.73	0.12	0.08	all *p* > 0.05
	AD	72453.30	9273.70	72371.37	9155.49	81.93	545.67		25.88	1.34	25.87	1.33	0.01	0.10	
	HC	77708.63	8387.38	77694.00	8392.59	14.63	72.29		26.66	1.14	26.79	1.18	−0.12	0.68	
Parietal Left	iNPH	24024.05	21592.56	23551.29	21031.64	472.76	1414.00	iNPH>HC*	11.82	0.49	11.77	0.48	0.05	0.06	all *p* > 0.05
	AD	46883.11	7368.76	46790.07	7290.69	93.04	316.13		10.55	0.61	10.54	0.60	0.01	0.02	
	HC	52576.80	6192.32	52563.13	6199.92	13.67	54.74		11.12	0.46	11.08	0.52	0.04	0.20	
Temporal Left	iNPH	18552.15	20247.22	18508.36	20237.00	43.78	163.17	all *p* > 0.05	23.78	1.37	23.70	1.35	0.08	0.07	all *p* > 0.05
	AD	43225.59	7460.71	43214.41	7450.57	11.19	78.14		22.45	1.71	22.45	1.70	0.00	0.06	
	HC	50498.93	5080.39	50501.70	5069.62	−2.77	55.24		24.47	1.08	24.11	1.81	0.37	1.44	
Occipital Left	iNPH	6968.31	4692.93	6921.50	4736.47	46.81	190.65	all *p* > 0.05	7.70	0.42	7.70	0.41	0.00	0.03	all *p* > 0.05
	AD	21937.30	3412.64	21891.44	3420.34	45.85	197.02		7.40	0.33	7.40	0.33	0.01	0.04	
	HC	23544.13	2652.42	23538.57	2654.60	5.57	23.25		7.58	0.30	7.58	0.35	−0.01	0.15	
Cingulate Left	iNPH	3539.31	2722.25	3469.56	2687.10	69.75	136.51	iNPH>AD* iNPH>HC*	9.09	0.52	9.08	0.55	0.00	0.14	all *p* > 0.05
	AD	8864.07	1590.36	8845.22	1581.45	18.85	64.79		9.24	0.65	9.24	0.65	0.00	0.05	
	HC	9556.57	1501.67	9552.33	1504.06	4.23	17.66		9.41	0.45	9.49	0.50	−0.07	0.29	

Delta values for each group have been calculated averaging the difference between pre and post cortical thickness and volume values for each subject. ^*^p ≤ 0.05, ^**^p ≤ 0.01, ^***^p ≤ 0.0001.

Spearman correlations showed that, in the left and right temporal lobe, cortical thickness changes significantly correlated with the Radscale score (rho = 0.61/60, *p* = 0.01), and the left temporal lobe volume also correlated with the callosal angle in isolation (rho = −0.53,*p* = 0.03). We also found a significant correlation between DESH scores and the change in volume of the right occipital lobe (rho = −0.51, *p* = 0.04).

The additional evaluations of FreeSurfer’s accuracy using HD BET in the pre-processing step revealed 7 segmentation failures and the necessity of multiple manual edits in 11 scans. Similarly, employing a combination of FLAIR and T1 images also led to seven segmentation failures and required manual corrections in five outputs.

## Discussion

The higher proportion of scans requiring correction within our sample of iNPH patients relative to the AD and HC groups underlines the importance of conducting and reporting this quality check in this group – which is not consistently done (Cogswell et al., 2021).

Whilst we acknowledge that the overall effect of the correction in these data is minor, it is important to note that this is a relatively small sample size and that we employed a conservative manual correction approach to mitigate the risk of bias associated with human judgement; the effect of this correction process might become substantial enough to influence results significantly when conducting larger studies. Our findings also reveal a statistically higher proportion of volume alterations attributed to manual edits in individuals with iNPH compared to both healthy controls and patients with AD. Changes in cortical thickness were in part correlated with the severity of radiological features of iNPH and underline the importance of exercising caution when using FreeSurfer with severe hydrocephalus. It is important to underline that one significant limitation of this study is the subjectivity of the visual inspections and manual corrections, which are prone to human error. However, we have followed the methodology and guidelines provided by the developers to mitigate bias and maximise consistency in our approach.^5^

The challenge for the field lies in establishing brain biomarkers that can differentiate between iNPH and other dementia types with overlapping clinical presentations and radiological features, such as ventriculomegaly, in order to identify patients to target with therapeutic shunting. Previous studies have demonstrated abnormal cortical thickening in the parietal lobe, and in the high convexity of the frontal, parietal, and occipital lobes in iNPH patients compared to healthy individuals and patients with Alzheimer’s disease (Moore et al., 2012; Kang et al., 2020; Bianco et al., 2022). Studies have suggested that cortical thickening may be characteristic of iNPH and related to the ventricular expansion, which leads to compression and stretching of the brain tissue, which may then reduce the cerebrospinal fluid space in the high convexity regions (Kang et al., 2020; Han et al., 2022). We cautiously suggest that increased cortical thickness and tightness of the high-convexity space increase the likelihood of FreeSurfer failing to delineate the pia from the dura and hence erroneously classifying extra voxels to grey matter. If not corrected, these inaccuracies may provide even further and exaggerate evidence of increased cortical thickness and volumes in these areas. Interestingly, segmentation errors did not affect the white matter surface. FreeSurfer’s failures seems to specifically impact the delineation of the pial surface. Since this is measured as the interface between the pial and the CSF, these inaccuracies could arise from the reduced CSF space and the tight high-convexity regions resulting from ventricular expansion.

In light of the challenges discussed above, we propose that researchers consider the likely lengthy process of manual correction that is required when using FreeSurfer in this clinical group and encourage the reporting of the completion of this step so that readers can have confidence in any associated results. However, there is a need for further, large-scale iNPH studies to reliably identify disease-specific biomarkers. In this case, conducting laborious manual corrections which can take several hours per subject (Lotan et al., 2022) may be unfeasible and introduce the likelihood of bias, especially given the challenges in blinding raters to the clinical group each scan comes from, given such apparent structural abnormalities.

With this in mind, alternative automatised software and analysis techniques with superior accuracy have been developed and may be preferential (Carass et al., 2017; Shao et al., 2019; Billot et al., 2023). Nevertheless, as shown above, FreeSurfer is still being widely used in current studies. This may be due to some limitations of these alternative tools. These in fact do not always provide segmentation of the individual compartments of the ventricles or are validated in small subsamples of iNPH patients (Shiee et al., 2011; Roy et al., 2015; Shao et al., 2019), do not improve the required processing time relative to FreeSurfer (Ellingsen et al., 2016), are not always freely available (Shao et al., 2019) or easily accessible as FreeSurfer (Ellingsen et al., 2016), or need manual delineation of new atlases when employed with new scanners (Roy et al., 2015).

## Data availability statement

The raw data supporting the conclusions of this article will be made available by the authors upon reasonable request.

## Ethics statement

The studies involving humans were approved by the Health Research Authority’s London-Surrey Borders Research Ethics Committee (19/LO/0102) and the Health Research Authority’s London-Central Research Ethics Committee (18/LO/0249). All participants gave written and/or electronic consent. The studies were conducted in accordance with the local legislation and institutional requirements. The participants provided their written informed consent to participate in this study.

## Author contributions

MG: Conceptualization, Data curation, Formal analysis, Investigation, Methodology, Project administration, Writing – original draft, Writing – review & editing, Visualization. MD: Data curation, Visualization, Writing – review & editing, Resources. MK: Data curation, Writing – review & editing, Resources. AG: Data curation, Writing – review & editing, Investigation, Resources. TP: Supervision, Writing – review & editing, Methodology. AH: Supervision, Writing – review & editing. DS: Supervision, Writing – review & editing, Funding acquisition, Resources. PM: Conceptualization, Investigation, Supervision, Writing – review & editing. CC: Conceptualization, Investigation, Methodology, Project administration, Resources, Supervision, Writing – original draft.

## Footnotes

^1^http://surfer.nmr.mgh.harvard.edu/

^2^https://surfer.nmr.mgh.harvard.edu/fswiki/FsTutorial/TroubleshootingData

^3^https://surfer.nmr.mgh.harvard.edu/fswiki/CorticalParcellation

^4^https://surfer.nmr.mgh.harvard.edu/fswiki/recon-all#UsingT2orFLAIRdatatoimprovepialsurfaces

^5^https://surfer.nmr.mgh.harvard.edu/fswiki/FsTutorial/PialEditsV6.0
