# Supplementary material for: Methodological challenges of measuring brain volumes and cortical thickness in idiopathic normal pressure hydrocephalus with a surface-based approach
Source: Front Neurosci. 2024 Jul 19;18:1366029. doi: 10.3389/fnins.2024.1366029 (PMC11295655; doi:10.3389/fnins.2024.1366029)
Supplement: Supplementary file 1 [file Table_1.docx]

Supplementary table 1. Values of cortical thickness and volumes before and after manual correction for all the 34 sub-regions calculated by Freesurfer, and between-group comparisons of the delta values (FDR corrected). Delta values for each group are a mean of the subjects-level delta between pre and post cortical thickness and volume values.

|  |  | **Volume (mm^3^ )** | | | | | | | **Cortical Thickness (mm)** | | | | | | |
| --- | --- | --- | --- | --- | --- | --- | --- | --- | --- | --- | --- | --- | --- | --- | --- |
|  |  | Pre correction | | Post correction | | Delta | | | Pre correction | | Post correction | | Delta | | |
| Region | Group | Mean | std | Mean | std | Mean | std | Group differences (p) | Mean | std | Mean | std | Mean | std | Group differences (p) |
| Bankssts | iNPH | 3814.19 | 512.50 | 3795.06 | 485.28 | 19.13 | 102.39 | all p>0.05 | 4.83 | 0.34 | 4.78 | 0.21 | 0.04 | 0.25 | all p>0.05 |
|  | AD | 3731.37 | 691.65 | 3725.74 | 693.36 | 5.63 | 25.17 |  | 4.54 | 0.34 | 4.56 | 0.34 | -0.01 | 0.08 |  |
|  | HC | 4262.07 | 585.41 | 4261.83 | 587.67 | 0.23 | 13.82 |  | 4.86 | 0.25 | 4.88 | 0.25 | -0.02 | 0.07 |  |
| Caudal anterior cingulate | iNPH | 2430.44 | 668.20 | 2401.81 | 625.91 | 28.63 | 89.59 | all p>0.05 | 4.32 | 0.32 | 4.35 | 0.32 | -0.02 | 0.37 | all p>0.05 |
|  | AD | 3314.44 | 517.59 | 3311.85 | 519.22 | 2.59 | 16.91 |  | 4.64 | 0.43 | 4.58 | 0.43 | 0.06 | 0.16 |  |
|  | HC | 3400.97 | 804.40 | 3403.60 | 798.39 | -2.63 | 31.45 |  | 4.55 | 0.33 | 4.56 | 0.33 | -0.01 | 0.08 |  |
| Caudal middle frontal | iNPH | 11340.44 | 2837.20 | 10839.88 | 2387.82 | 500.56 | 629.55 | NPH>AD*** NPH>HC*** | 5.09 | 0.30 | 5.02 | 0.22 | 0.07 | 0.21 | all p>0.05 |
|  | AD | 9738.22 | 2273.54 | 9679.93 | 2192.00 | 58.30 | 241.97 |  | 4.60 | 0.32 | 4.60 | 0.30 | 0.00 | 0.08 |  |
|  | HC | 10764.90 | 1912.62 | 10758.43 | 1917.55 | 6.47 | 28.67 |  | 4.75 | 0.22 | 4.76 | 0.22 | -0.01 | 0.04 |  |
| Cuneus | iNPH | 6270.44 | 1215.81 | 6266.94 | 1199.04 | 3.50 | 105.36 | all p>0.05 | 3.91 | 0.22 | 3.90 | 0.19 | 0.02 | 0.21 | all p>0.05 |
|  | AD | 6609.19 | 1413.97 | 6592.15 | 1414.15 | 17.03 | 65.83 |  | 3.77 | 0.19 | 3.76 | 0.19 | 0.01 | 0.04 |  |
|  | HC | 6530.83 | 1185.47 | 6537.07 | 1182.74 | -6.23 | 23.91 |  | 3.78 | 0.17 | 3.79 | 0.17 | -0.01 | 0.03 |  |
| Enthorhinal | iNPH | 3468.81 | 562.50 | 3370.50 | 518.45 | 98.31 | 210.92 | NPH>AD** NPH>HC** | 5.87 | 0.47 | 5.82 | 0.56 | 0.04 | 0.30 | all p>0.05 |
|  | AD | 2987.78 | 865.89 | 2994.15 | 850.86 | -6.37 | 78.79 |  | 5.40 | 0.83 | 5.43 | 0.81 | -0.03 | 0.31 |  |
|  | HC | 3577.63 | 495.83 | 3578.30 | 487.05 | -0.67 | 26.12 |  | 6.28 | 0.40 | 6.26 | 0.40 | 0.02 | 0.07 |  |
| Fusiform | iNPH | 17534.69 | 1467.56 | 17353.63 | 1570.20 | 181.06 | 535.51 | all p>0.05 | 5.27 | 0.31 | 5.24 | 0.27 | 0.04 | 0.23 | all p>0.05 |
|  | AD | 16193.22 | 2393.44 | 16153.93 | 2386.66 | 39.30 | 136.45 |  | 4.93 | 0.31 | 4.96 | 0.31 | -0.03 | 0.13 |  |
|  | HC | 18651.93 | 2599.03 | 18650.50 | 2601.02 | 1.43 | 10.80 |  | 5.30 | 0.17 | 5.30 | 0.17 | 0.00 | 0.00 |  |
| Inferior parietal | iNPH | 23811.13 | 3644.31 | 23732.50 | 3617.63 | 78.63 | 181.85 | all p>0.05 | 4.93 | 0.30 | 4.86 | 0.21 | 0.06 | 0.21 | all p>0.05 |
|  | AD | 20371.52 | 3601.60 | 20321.15 | 3567.09 | 50.37 | 246.59 |  | 4.31 | 0.30 | 4.34 | 0.30 | -0.02 | 0.13 |  |
|  | HC | 24675.03 | 3495.25 | 24657.00 | 3501.91 | 18.03 | 71.01 |  | 4.65 | 0.22 | 4.67 | 0.22 | -0.02 | 0.09 |  |
| Inferior temporal | iNPH | 19297.69 | 1978.59 | 19159.88 | 1962.59 | 137.81 | 356.53 | NPH>AD** NPH>HC** | 5.48 | 0.28 | 5.43 | 0.21 | 0.04 | 0.24 | all p>0.05 |
|  | AD | 16951.74 | 2733.39 | 16958.44 | 2731.84 | -6.70 | 47.97 |  | 4.96 | 0.28 | 4.99 | 0.27 | -0.03 | 0.18 |  |
|  | HC | 20006.17 | 2315.90 | 20009.33 | 2301.97 | -3.17 | 47.38 |  | 5.30 | 0.25 | 5.30 | 0.25 | -0.01 | 0.05 |  |
| Isthmus cingulate | iNPH | 4448.63 | 985.39 | 4219.81 | 780.67 | 228.81 | 637.46 | NPH>AD* NPH>HC* | 4.22 | 0.23 | 4.25 | 0.18 | -0.03 | 0.21 | all p>0.05 |
|  | AD | 4694.04 | 869.83 | 4679.48 | 852.30 | 14.56 | 52.32 |  | 4.21 | 0.37 | 4.22 | 0.35 | -0.01 | 0.07 |  |
|  | HC | 4899.23 | 662.31 | 4906.90 | 661.92 | -7.67 | 29.74 |  | 4.41 | 0.27 | 4.40 | 0.28 | 0.01 | 0.04 |  |
| Lateral occipital | iNPH | 23889.69 | 3766.83 | 23872.00 | 3554.79 | 17.69 | 472.01 | all p>0.05 | 4.48 | 0.31 | 4.44 | 0.21 | 0.04 | 0.23 | all p>0.05 |
|  | AD | 21928.70 | 3369.20 | 21884.56 | 3367.91 | 44.15 | 174.84 |  | 4.16 | 0.25 | 4.17 | 0.24 | -0.01 | 0.07 |  |
|  | HC | 24687.80 | 3276.67 | 24687.40 | 3277.23 | 0.40 | 4.22 |  | 4.35 | 0.22 | 4.36 | 0.22 | -0.01 | 0.07 |  |
| Lateral orbitofrontal | iNPH | 13225.69 | 1611.04 | 13163.44 | 1623.36 | 62.25 | 148.07 | all p>0.05 | 4.82 | 0.19 | 4.83 | 0.12 | -0.01 | 0.17 | all p>0.05 |
|  | AD | 12942.96 | 1980.93 | 12954.59 | 1989.24 | -11.63 | 136.78 |  | 4.89 | 0.26 | 4.87 | 0.27 | 0.02 | 0.06 |  |
|  | HC | 13829.40 | 1552.32 | 13834.00 | 1541.15 | -4.60 | 54.37 |  | 5.00 | 0.26 | 5.00 | 0.26 | 0.00 | 0.02 |  |
| Lingual | iNPH | 13098.63 | 2504.56 | 13061.56 | 2474.05 | 37.06 | 353.45 | all p>0.05 | 4.00 | 0.26 | 3.98 | 0.21 | 0.02 | 0.16 | all p>0.05 |
|  | AD | 13139.81 | 2170.63 | 13126.85 | 2186.66 | 12.96 | 102.12 |  | 3.89 | 0.19 | 3.91 | 0.18 | -0.02 | 0.07 |  |
|  | HC | 13528.43 | 1597.62 | 13533.17 | 1597.37 | -4.73 | 36.53 |  | 3.98 | 0.15 | 3.97 | 0.15 | 0.00 | 0.01 |  |
| Medial orbitofrontal | iNPH | 9708.63 | 988.92 | 9692.50 | 957.97 | 16.13 | 192.00 | all p>0.05 | 4.48 | 0.22 | 4.45 | 0.17 | 0.02 | 0.18 | all p>0.05 |
|  | AD | 9604.70 | 1216.04 | 9607.19 | 1223.66 | -2.48 | 45.79 |  | 4.55 | 0.32 | 4.56 | 0.31 | 0.00 | 0.09 |  |
|  | HC | 10099.03 | 932.78 | 10109.77 | 924.45 | -10.73 | 44.69 |  | 4.64 | 0.25 | 4.65 | 0.23 | -0.01 | 0.06 |  |
| Middle temporal | iNPH | 19885.25 | 2052.42 | 19814.00 | 2054.95 | 71.25 | 235.74 | all p>0.05 | 5.39 | 0.27 | 5.35 | 0.21 | 0.04 | 0.17 | all p>0.05 |
|  | AD | 17631.93 | 3378.74 | 17610.70 | 3372.55 | 21.22 | 64.39 |  | 4.92 | 0.38 | 4.96 | 0.36 | -0.04 | 0.21 |  |
|  | HC | 20652.40 | 2237.37 | 20649.67 | 2237.96 | 2.73 | 14.97 |  | 5.32 | 0.23 | 5.33 | 0.24 | 0.00 | 0.03 |  |
| Parahippocampal | iNPH | 4026.56 | 621.43 | 3955.81 | 618.92 | 70.75 | 135.74 | NPH>AD** NPH>HC** | 4.98 | 0.58 | 4.99 | 0.42 | 0.00 | 0.33 | all p>0.05 |
|  | AD | 3380.74 | 524.90 | 3372.78 | 516.95 | 7.96 | 29.40 |  | 4.80 | 0.57 | 4.84 | 0.49 | -0.04 | 0.24 |  |
|  | HC | 3912.30 | 448.10 | 3911.87 | 447.77 | 0.43 | 2.03 |  | 5.26 | 0.35 | 5.24 | 0.36 | 0.02 | 0.10 |  |
| Paracentral | iNPH | 7331.19 | 1043.73 | 7257.75 | 1046.49 | 73.44 | 115.13 | NPH>AD*** NPH>HC*** | 4.74 | 0.29 | 4.72 | 0.21 | 0.02 | 0.26 | all p>0.05 |
|  | AD | 6720.07 | 731.65 | 6716.93 | 743.52 | 3.14 | 37.38 |  | 4.69 | 0.21 | 4.68 | 0.21 | 0.01 | 0.06 |  |
|  | HC | 6888.43 | 867.94 | 6896.97 | 857.83 | -8.53 | 40.48 |  | 4.62 | 0.21 | 4.63 | 0.22 | -0.01 | 0.12 |  |
| Pars opercularis | iNPH | 6977.50 | 878.89 | 6995.13 | 885.19 | -17.63 | 137.61 | all p>0.05 | 4.74 | 0.17 | 4.77 | 0.14 | 0.00 | 0.12 | all p>0.05 |
|  | AD | 7112.93 | 1087.16 | 7102.48 | 1076.53 | 10.44 | 55.37 |  | 4.73 | 0.30 | 4.72 | 0.30 | 0.01 | 0.06 |  |
|  | HC | 7475.10 | 1282.71 | 7481.27 | 1281.68 | -6.17 | 23.51 |  | 4.88 | 0.24 | 4.89 | 0.22 | -0.01 | 0.06 |  |
| Pars orbitalis | iNPH | 4501.50 | 576.57 | 4514.75 | 569.91 | -13.25 | 125.69 | all p>0.05 | 4.92 | 0.25 | 4.86 | 0.22 | 0.06 | 0.15 | NPH>AD** NPH>HC** |
|  | AD | 4426.48 | 664.68 | 4424.26 | 656.74 | 2.22 | 28.85 |  | 4.94 | 0.33 | 4.96 | 0.33 | -0.02 | 0.05 |  |
|  | HC | 4754.33 | 575.23 | 4752.93 | 578.16 | 1.40 | 16.38 |  | 5.06 | 0.33 | 5.07 | 0.32 | 0.00 | 0.03 |  |
| Pars triangularis | iNPH | 6568.19 | 710.38 | 6573.69 | 725.21 | -5.50 | 108.18 | all p>0.05 | 4.58 | 0.11 | 4.55 | 0.12 | 0.03 | 0.13 | all p>0.05 |
|  | AD | 6521.48 | 1069.49 | 6522.44 | 1074.75 | -0.96 | 36.74 |  | 4.43 | 0.29 | 4.44 | 0.28 | 0.00 | 0.05 |  |
|  | HC | 7107.50 | 1260.04 | 7105.93 | 1260.44 | 1.57 | 22.22 |  | 4.58 | 0.21 | 4.58 | 0.21 | -0.01 | 0.02 |  |
| Pericalcarine | iNPH | 4060.31 | 1172.12 | 4056.63 | 1140.55 | 3.69 | 63.87 | all p>0.05 | 3.17 | 0.25 | 3.15 | 0.19 | 0.02 | 0.24 | all p>0.05 |
|  | AD | 4731.22 | 1205.91 | 4725.15 | 1207.66 | 6.07 | 77.97 |  | 3.26 | 0.20 | 3.26 | 0.19 | -0.01 | 0.06 |  |
|  | HC | 4442.43 | 903.60 | 4444.43 | 901.54 | -2.00 | 24.58 |  | 3.25 | 0.21 | 3.26 | 0.20 | -0.01 | 0.04 |  |
| Postcentral | iNPH | 18962.56 | 2473.21 | 18526.25 | 2549.58 | 436.31 | 293.59 | NPH>AD*** NPH>HC*** | 4.23 | 0.20 | 4.20 | 0.18 | 0.03 | 0.15 | all p>0.05 |
|  | AD | 16886.78 | 2127.00 | 16862.74 | 2093.68 | 24.04 | 154.11 |  | 3.92 | 0.22 | 3.93 | 0.22 | 0.00 | 0.02 |  |
|  | HC | 17741.70 | 2346.13 | 17742.70 | 2345.21 | -1.00 | 7.59 |  | 4.04 | 0.24 | 4.04 | 0.24 | -0.01 | 0.02 |  |
| Posterior cingulate | iNPH | 3945.19 | 1285.11 | 3797.19 | 936.19 | 148.00 | 667.27 | all p>0.05 | 4.43 | 0.31 | 4.52 | 0.21 | -0.09 | 0.29 | all p>0.05 |
|  | AD | 5334.48 | 803.26 | 5326.96 | 804.26 | 7.52 | 31.80 |  | 4.42 | 0.24 | 4.41 | 0.23 | 0.01 | 0.05 |  |
|  | HC | 5574.53 | 708.13 | 5571.20 | 708.83 | 3.33 | 14.41 |  | 4.49 | 0.18 | 4.50 | 0.17 | 0.00 | 0.04 |  |
| Precentral | iNPH | 24508.75 | 3124.56 | 23970.50 | 2912.68 | 538.25 | 454.63 | NPH>AD*** NPH>HC*** | 4.78 | 0.28 | 4.77 | 0.21 | 0.01 | 0.17 | all p>0.05 |
|  | AD | 23612.52 | 2633.54 | 23569.41 | 2579.12 | 43.11 | 153.81 |  | 4.77 | 0.23 | 4.77 | 0.22 | 0.00 | 0.06 |  |
|  | HC | 24418.07 | 2663.43 | 24411.30 | 2667.74 | 6.77 | 28.85 |  | 4.80 | 0.25 | 4.80 | 0.25 | -0.01 | 0.03 |  |
| Precuneus | iNPH | 17424.19 | 2304.12 | 17386.94 | 2255.47 | 41.13 | 145.38 | all p>0.05 | 4.76 | 0.25 | 4.74 | 0.17 | 0.02 | 0.19 | all p>0.05 |
|  | AD | 17428.06 | 2306.55 | 16588.22 | 2725.95 | 6.26 | 85.48 |  | 4.33 | 0.26 | 4.34 | 0.26 | -0.01 | 0.03 |  |
|  | HC | 18527.23 | 2422.65 | 18536.93 | 2437.79 | -9.70 | 46.31 |  | 4.53 | 0.18 | 4.54 | 0.17 | -0.01 | 0.05 |  |
| Rostral anterior cingulate | iNPH | 3621.69 | 849.31 | 3585.38 | 843.07 | 36.31 | 60.58 | NPH>AD** NPH>HC** | 5.09 | 0.22 | 5.08 | 0.43 | 0.01 | 0.44 | all p>0.05 |
|  | AD | 3903.85 | 748.49 | 3906.00 | 739.68 | -2.15 | 35.09 |  | 5.21 | 0.39 | 5.17 | 0.37 | 0.04 | 0.18 |  |
|  | HC | 4471.67 | 823.35 | 4467.90 | 826.09 | 3.77 | 17.54 |  | 5.30 | 0.41 | 5.33 | 0.38 | -0.02 | 0.08 |  |
| Rostral middle frontal | iNPH | 26921.44 | 3474.42 | 26668.19 | 3476.23 | 253.25 | 507.53 | NPH>AD** NPH>HC** | 4.60 | 0.21 | 4.55 | 0.17 | 0.04 | 0.15 | all p>0.05 |
|  | AD | 25278.00 | 4146.09 | 25288.59 | 4142.21 | -10.60 | 84.53 |  | 4.35 | 0.27 | 4.36 | 0.27 | -0.01 | 0.06 |  |
|  | HC | 27557.43 | 4020.86 | 27575.07 | 4012.03 | -17.63 | 67.12 |  | 4.44 | 0.23 | 4.45 | 0.22 | -0.01 | 0.05 |  |
| Superior frontal | iNPH | 40774.06 | 5977.14 | 39966.94 | 5840.33 | 807.13 | 635.98 | NPH>AD*** NPH>HC*** | 5.26 | 0.30 | 5.20 | 0.21 | 0.05 | 0.24 | all p>0.05 |
|  | AD | 36087.33 | 5131.18 | 36072.00 | 5092.03 | 15.33 | 254.85 |  | 4.85 | 0.27 | 4.85 | 0.26 | 0.00 | 0.07 |  |
|  | HC | 39589.83 | 4291.38 | 39563.90 | 4221.85 | 25.93 | 106.36 |  | 4.98 | 0.22 | 4.99 | 0.22 | -0.02 | 0.07 |  |
| Superior temporal | iNPH | 22140.19 | 3583.87 | 25356.56 | 3603.78 | -3216.38 | 2497.69 | NPH>AD*** NPH>HC*** | 4.73 | 0.31 | 4.69 | 0.20 | 0.01 | 0.11 | all p>0.05 |
|  | AD | 23262.22 | 3648.00 | 23264.63 | 3660.68 | -2.41 | 78.34 |  | 4.09 | 0.27 | 4.10 | 0.26 | -0.02 | 0.11 |  |
|  | HC | 25205.20 | 3167.05 | 25196.03 | 3167.32 | -2.60 | 41.89 |  | 4.25 | 0.21 | 4.27 | 0.23 | 0.00 | 0.01 |  |
| Superior parietal | iNPH | 24793.56 | 3006.70 | 21451.38 | 2048.48 | 3342.19 | 2512.82 | NPH>AD*** NPH>HC*** | 5.08 | 0.22 | 5.07 | 0.22 | 0.04 | 0.26 | all p>0.05 |
|  | AD | 20000.48 | 3142.83 | 19991.41 | 3140.97 | 9.07 | 42.12 |  | 4.85 | 0.31 | 4.87 | 0.28 | 0.00 | 0.04 |  |
|  | HC | 22383.57 | 3341.69 | 22386.17 | 3335.92 | 9.17 | 37.81 |  | 5.24 | 0.26 | 5.25 | 0.25 | -0.02 | 0.10 |  |
| Supramarginal | iNPH | 18546.88 | 2007.18 | 18470.88 | 1979.36 | 76.00 | 208.32 | all p>0.05 | 4.79 | 0.20 | 4.76 | 0.19 | 0.03 | 0.14 | all p>0.05 |
|  | AD | 18154.07 | 3222.81 | 18107.00 | 3232.64 | 47.07 | 177.26 |  | 4.54 | 0.25 | 4.54 | 0.23 | -0.01 | 0.07 |  |
|  | HC | 19427.33 | 2803.54 | 19432.87 | 2797.13 | -5.53 | 26.46 |  | 4.78 | 0.25 | 4.80 | 0.24 | -0.02 | 0.06 |  |
| Frontal pole | iNPH | 2125.56 | 240.19 | 2109.88 | 247.91 | 15.69 | 57.48 | all p>0.05 | 5.11 | 0.32 | 5.06 | 0.26 | 0.04 | 0.27 | all p>0.05 |
|  | AD | 2125.63 | 311.39 | 2125.89 | 309.51 | -0.26 | 28.45 |  | 5.09 | 0.38 | 5.07 | 0.39 | 0.02 | 0.08 |  |
|  | HC | 2162.60 | 339.69 | 2163.17 | 347.37 | -0.57 | 34.86 |  | 5.25 | 0.49 | 5.29 | 0.47 | -0.03 | 0.14 |  |
| Temporal pole | iNPH | 5027.50 | 676.29 | 4890.94 | 552.97 | 136.56 | 165.02 | NPH>AD*** NPH>HC*** | 6.64 | 0.53 | 6.67 | 0.35 | -0.03 | 0.42 | all p>0.05 |
|  | AD | 4656.59 | 955.03 | 4643.07 | 939.24 | 13.50 | 43.31 |  | 6.33 | 0.69 | 6.34 | 0.69 | -0.01 | 0.18 |  |
|  | HC | 5147.47 | 715.78 | 5154.47 | 717.12 | -7.00 | 27.50 |  | 6.98 | 0.47 | 6.97 | 0.48 | 0.01 | 0.08 |  |
| Transverse temporal | iNPH | 1927.63 | 196.04 | 1949.81 | 212.46 | -22.19 | 70.73 | all p>0.05 | 4.53 | 0.36 | 4.48 | 0.29 | 0.05 | 0.22 | all p>0.05 |
|  | AD | 1884.19 | 357.90 | 1886.85 | 357.27 | -2.67 | 7.02 |  | 4.56 | 0.26 | 4.59 | 0.22 | -0.03 | 0.14 |  |
|  | HC | 2001.63 | 377.20 | 2004.30 | 375.97 | -2.67 | 12.53 |  | 4.69 | 0.34 | 4.70 | 0.34 | -0.01 | 0.07 |  |
| Insula | iNPH | 12562.56 | 1418.87 | 12588.63 | 1485.29 | -26.06 | 161.98 | all p>0.05 | 5.27 | 0.36 | 5.25 | 0.25 | 0.02 | 0.21 | all p>0.05 |
|  | AD | 12356.85 | 1558.60 | 12340.04 | 1557.15 | 16.80 | 134.98 |  | 5.38 | 0.34 | 5.40 | 0.33 | -0.02 | 0.06 |  |
|  | HC | 13173.50 | 1474.15 | 375.97 | 1469.68 | -5.07 | 19.50 |  | 5.67 | 0.33 | 5.66 | 0.34 | 0.01 | 0.03 |  |

*p<0.05, ** p<0.01, ***p<0.001
